# Novel Anode Catalyst for Direct Methanol Fuel Cells

**DOI:** 10.1155/2014/547604

**Published:** 2014-04-27

**Authors:** S. Basri, S. K. Kamarudin, W. R. W. Daud, Z. Yaakob, A. A. H. Kadhum

**Affiliations:** ^1^Fuel Cell Institute, Universiti Kebangsaan Malaysia (UKM), 43600 Bangi, Selangor, Malaysia; ^2^Department of Chemical and Process Engineering, Universiti Kebangsaan Malaysia (UKM), 43600 Bangi, Selangor, Malaysia

## Abstract

PtRu catalyst is a promising anodic catalyst for direct methanol fuel cells (DMFCs) but the slow reaction kinetics reduce the performance of DMFCs. Therefore, this study attempts to improve the performance of PtRu catalysts by adding nickel (Ni) and iron (Fe). Multiwalled carbon nanotubes (MWCNTs) are used to increase the active area of the catalyst and to improve the catalyst performance. Electrochemical analysis techniques, such as energy dispersive X-ray spectrometry (EDX), X-ray diffraction (XRD), field emission scanning electron microscopy (FESEM), and X-ray photoelectron spectroscopy (XPS), are used to characterize the kinetic parameters of the hybrid catalyst. Cyclic voltammetry (CV) is used to investigate the effects of adding Fe and Ni to the catalyst on the reaction kinetics. Additionally, chronoamperometry (CA) tests were conducted to study the long-term performance of the catalyst for catalyzing the methanol oxidation reaction (MOR). The binding energies of the reactants and products are compared to determine the kinetics and potential surface energy for methanol oxidation. The FESEM analysis results indicate that well-dispersed nanoscale (2–5 nm) PtRu particles are formed on the MWCNTs. Finally, PtRuFeNi/MWCNT improves the reaction kinetics of anode catalysts for DMFCs and obtains a mass current of 31 A g^−1^ catalyst.

## 1. Introduction

Most fuel cell catalytic electrodes, including DMFC anodes, are made of either platinum or platinum-based materials [1]. However, many problems that prevent the commercialization of DMFCs remain unsolved, such as poor performance, catalyst instability, methanol crossover, and thermal and water management [2–4]. Thus, this study focused on the poor methanol oxidation kinetics, which is partially attributed to catalyst poisoning [5, 6] of the anode by reaction intermediates such as CO [7]. During methanol oxidation, strongly adsorbed CO blocks Pt sites required for the oxidative adsorption of methanol. The enthalpy of adsorption is tuned by varying the alloy composition (compositional tuning) or electrode potential (Stark tuning). Such variations affect the interactions between CO molecular orbitals (MOs) and metal substrate bands. Changes in the electronic structure of mixed-metal catalysts are monitored by polarization-modulated infrared reflection adsorption spectroscopy (PM-IRAS) of adsorbed CO [8, 9]. Therefore, to improve DMFC efficiency, anode electrocatalysts that enhance methanol oxidation activity and minimize CO poisoning must be considered.

The high reactivity of Pt makes it a suitable anode electrocatalyst for DMFCs. However, pure Pt is readily poisoned by carbon monoxide (CO), the main intermediate species formed by an indirect reaction during methanol oxidation. This indirect reaction occurs because less energy is required to form CO than CO_2_. Therefore, to avoid the formation of CO on Pt electrodes, binary or hybrid alloys of Pt, such as PtRu, PtSn [10], Pt-M [11], PtMO [12], PtPbMnO [13], and PtCo [14, 15], are generally employed as electrocatalytic materials on DMFC anodes. In DMFCs, bimetallic PtRu is the most widely used catalytic material because of its high electrocatalytic activity towards methanol oxidation at the anode. Ru improved the electronic properties of Pt to prevent the adoption of CO by decreasing the oxidation overpotential of the anode [16]. However, slow reaction kineticsis a significant limitation that reduces the performance and power output of DMFCs.

Thus, two major improvements are essential in catalyst layer design: the incorporation of Pt [17] or Pt-group [18, 19] nanoparticles and the impregnation or colloidal mixing of a high surface area carbon support with an ionomer. Several PtRu-metal groups have been considered as potential anode catalysts for DMFCs, including rhodium (Rh), iron (Fe), nickel (Ni), and cobalt (Co). Of these metal groups, Park et al. [20] studied the effect of adding nickel and rhodium. In their study, the PtRuRhNi catalyst obtained a high oxidation current of 100 mA cm^−1^ at high operating temperatures (70°C). Jeon et al. [21] studied several catalyst combinations, including Pt_45_Ru_45_Fe_10_/C, Pt_45_Ru_45_Co_10_/C, and Pt_45_Ru_45_Ni_10_/C. Among these catalysts, the Pt_45_Ru_45_Fe_10_/C catalyst exhibited the highest mass activity of 2.6 A g^−1^ catalyst, whereas the mass activities of the Pt_45_Ru_45_Co_10_/C and Pt_45_Ru_45_Ni_10_/C catalysts were 2.2 and 2.5 A g^−1^ catalyst, respectively. With respect to specific activity, the catalysts exhibited much higher activities of 110, 120, and 150 mA m^−2^ for the Fe-, Co-, and Ni-incorporated catalysts, respectively, than 88 mA m^−2^ of a commercial PtRu/C catalyst. Later, Jeon et al. [22] improved the PtRuFe/C catalyst and obtained mass and specific activities of 5.67 A g^−1^ catalyst and 177 mA m^−2^, respectively.

Wang et al. [26] also demonstrated via electrochemical impedance spectroscopy (EIS) that the performance of the PtRuNi/C catalyst for methanol electrooxidation was much better than that of the PtRu/C catalyst. This result was supported by Wang et al. [27], Ribeiro et al. [16], and Liu et al. [25] through their experimental studies on PtRu/C, in which they obtained mass activities of 3.7, 5.1, and 9.0 A g^−1^ catalyst, respectively. Jiang et al. [28] also demonstrated that a carbon-supported PtNi/C electrocatalyst showed enhanced mass activity for the methanol oxidation reaction compared to a Pt/C catalyst. In addition, the deposition of Pt nanoparticles on highly ordered carbon nanotubes (CNTs) has been reported as one of the reasons for the significantly increased methanol oxidation activity. In fact, the development of carbon catalyst support materials, such as carbon porous materials (CPM), carbon nanotubes (CNT), and activated carbon, and the addition of noble metals to catalysts are gaining considerable attention as two methods to improve methanol oxidation and DMFC efficiency [1].

Considering the great performance of Fe and Ni compared to other metals from previous studies, this study proposed a novel catalyst adopting Fe and Ni incorporated with PtRu using MWCNTs as a catalyst support. This study characterized the PtRuFeNi/MWCNT using electrochemical techniques such as energy dispersive X-ray (EDX) spectrometry, X-ray diffraction (XRD), X-ray photoelectron spectroscopy (XPS), and field emission scanning electron microscopy (FESEM). This study also investigated the effects of adding Fe and Ni atoms on the reaction kinetics using cyclic voltammetry (CV). This study also employed chronoamperometry tests to study the stability of the catalysts for the methanol oxidation reaction (MOR). Finally, this study determined and compared the mass activity and the specific activity of the proposed novel catalyst with other types of catalysts.

## 2. Experimental

### 2.1. Preparation of a Nanocatalyst

Industrial-grade MWCNTs were purified by refluxing in 60% HNO_3_ at 90°C for 2 h. The mixture was diluted with water, filtered, washed with excess deionized (DI) water, and dried overnight at 50°C in a vacuum oven. After purification, surface oxidation of MWCNTs was achieved by refluxing MWCNTs in 4 M H_2_SO_4_ and 4 M HNO_3_ at 90°C for 5 h. Finally, the treated MWCNTs were diluted with water, filtered, washed with excess DI water, and dried overnight at 50°C in a vacuum oven. PtRu nanoparticles were synthesized using NaBH_4_ as a reducing agent and deposited on MWCNTs along with H_2_PtCl_6_ (0.0065 mM) and RuCl_3_·6H_2_O (0.0065 mM) precursors in tetrahydrofuran (THF) [29]. After the reduction reaction, the mixture was stirred for 2 h at room temperature. Finally, the mixture was filtered, washed with excess DI water, and dried in a vacuum oven at 70°C for 30 min. Using the above procedure, PtRuFe/MWCNTs, PtRuNi/MWCNTs, and PtRuFeNi/MWCNTs (20 wt%) were also synthesized. For comparison, another method in which the stirring process was replaced with ultrasonic homogenization was also performed. The results from the two methods were compared in terms of particle size, distribution, and morphology using TEM.

### 2.2. Physical Characterization

The catalyst morphology was observed via TEM at 100 kV for samples diluted in the methanol solution. The sample was coated with gold before determining the elements contained in the sample using EDX, which was attached to the FESEM (SUPRA 55VP). Under vacuum conditions of 1 × 10^9^ torr, AES-XPS (Kratos Axis DLD) is used for chemical state analysis of atoms in the ten layers from the surface. One gram of sample was used for approximately 1–3 h of operation. XRD (D8 ADVANCE) was used for the physical characterization of the samples placed on a glass slip. The allowable scanning angle is between 5° and 90° degrees 2*θ*.

### 2.3. Electrochemical Analysis

A conventional three-electrode cell was constructed. A glassy carbon (GC) electrode with an area of 0.125 cm^2^ was used as the working electrode, a Pt wire was used as the counter electrode, and a saturated calomel electrode (SCE) was used as the reference electrode. The catalyst ink was prepared by first dispersing 7.2 mg of PtRu/MWCNTs and PtRu/MWCNTs (S) in 2.5 mL of ethanol with ultrasonication. Then, 0.5 mL of 0.1 wt% Nafion was added and dispersed with ultrasonication for 30 min. After sonication, 10 mL of the solution was added to the GC electrode. The electrode was dried at 70°C to yield a PtRu loading of 0.048 mg cm^−2^. CV experiments were performed in a solution containing 0.5 M H_2_SO_4_ and 1 M CH_3_OH at a scan rate of 50 mV s^−1^ [29] for in situ conditions. Ten consecutive runs were performed to obtain stable results.

## 3. Results and Discussion

**Figure 1:**
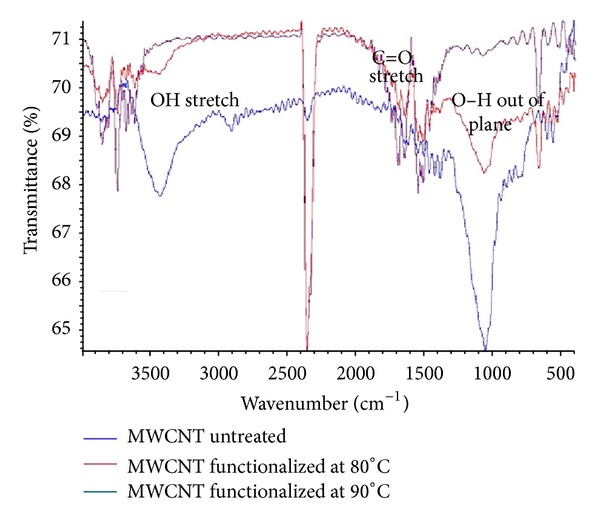
FT-IR analyses for pure MWCNTs before treatment and after treatment at 80°C and 90°C.

Initially, untreated MWCNTs contained only C–C bonds and no –OH bonds, as shown in Figure 1. Because C–C bonds are difficult to break, an oxidation process was used to facilitate the formation of –OH molecules on the MWCNTs. During catalyst synthesis, platinum and ruthenium from the H_2_PtCl_6_ and RuCl_3_·6H_2_O precursors take advantage of –OH bonds to provide better adsorption, which is called impregnation. The formation of –OH bonds on the MWCNTs was confirmed by FT-IR analysis of surface functional groups. The FT-IR results for MWCNTs refluxed at 90°C show the presence of –OH bonds, in contrast to the MWCNTs refluxed at 80°C. Therefore, temperature is a significant parameter for –OH bond formation. FT-IR bands correlating to –OH functional groups were observed at 3400–3600 cm^−1^, 1400 cm^−1^, and 1000 cm^−1^. MWCNTs treated at temperatures lower than 80°C did not exhibit obvious –OH stretching or in-plane bending. In contrast, MWCNTs treated at 90°C exhibited transmittance at all wavenumbers consistent with –OH stretching and both in-plane and out-of-plane bending. The water in the fuel cell is oxidized to a hydroxy radical via the following reaction: H_2_O → OH^•^ + H^+^ + e^−^. The hydroxy radical then oxidizes carbon monoxide to produce carbon dioxide, which is released from the surface as a gas: CO + OH^•^ → CO_2_ + H^+^ + e. It was also found that COOH may occur in the wavenumber range from 3400 to 2400 cm^−1^, which is very broad and overlaps with C–H and O–H stretching. However, this study focuses on the existence of OH after the oxidation process to provide better adsorption.

**Figure 2:**
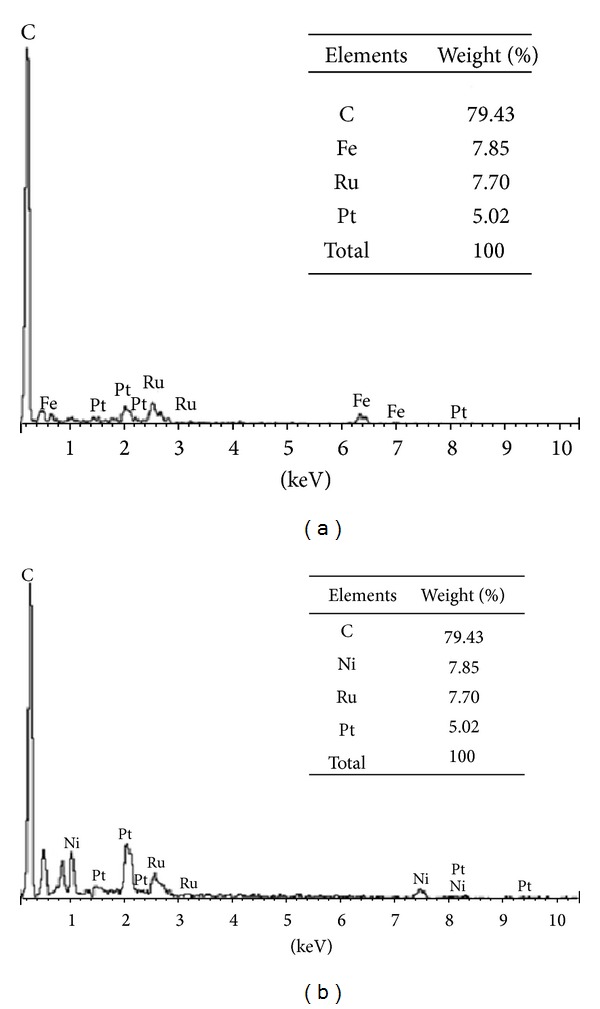
EDX results for MWCNT-based nanocatalyst with the addition of (a) iron and (b) nickel.

**Figure 3:**
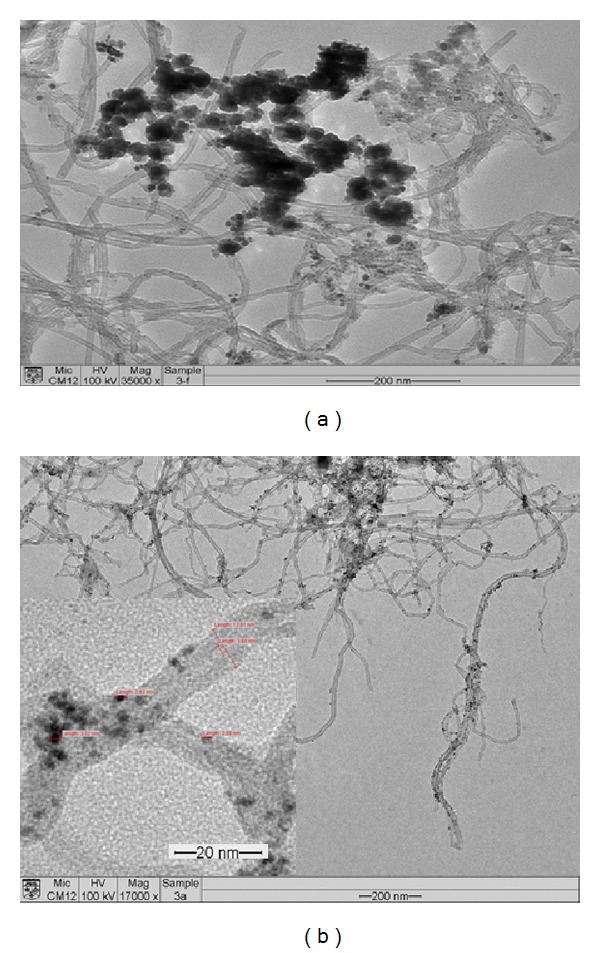
Morphologies of (a) PtRuFeNi/MWCNT and (b) PtRu/MWCNT.

Energy dispersive X-ray (EDX) spectrometry was used to quantify the atomic composition of the nanocatalysts. Figures 2(a) and 2(b) present the EDX results for nanocatalysts synthesized using the reduction method. From the graph, the atomic composition of the nanocatalysts was observed to be either 5% or 15% platinum. To increase the performance and reduce cost, nickel and iron were added to the nanocatalysts.  Figure 3 presents the surface morphologies of PtRu/MWCNTs and PtRuFeNi/MWCNTs after synthesis, which indicates that they are well distributed.

**Figure 4:**
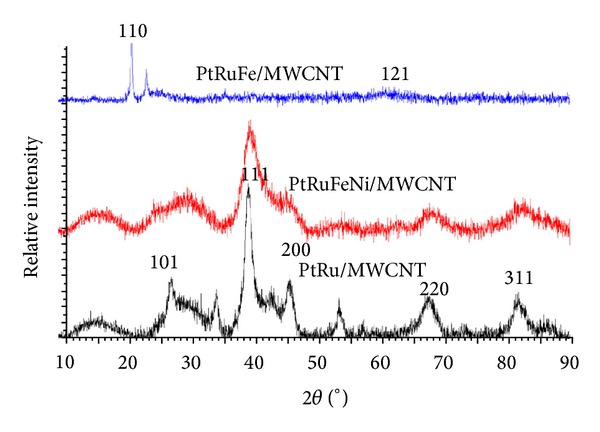
XRD patterns of PtRu/MWCNT, PtRuFeNi/MWCNT, and PtRuFe/MWCNT.

The X-ray diffraction (XRD) results presented in Figure 4 indicate that all prepared metal catalysts presented a typical face-centered cubic (fcc) crystallographic structure. Compared to the PtRuFeNi/MWCNT catalyst, there was a slight shift in the X-ray diffraction peaks of the PtRu/MWCNT catalyst to higher Bragg angles. This shift indicates that bimetallic interactions or alloying occurred in the catalyst. The small peak observed at 40° for the PtRuFe/MWCNT sample is attributed to Pt (111). The weak intensity and broad peak width indicate high metallic dispersion in the prepared sample.

The particle size was calculated using the Debye-Scherrer equation [23] with data from the XRD analysis as follows:

(1) $$\begin{array}{c} \text{Particle}\;\text{Size}=\frac{k\propto }{B\cos\theta }, \end{array}$$

where *k* = 0.98 and *α* = 0.154 nm.

Table 1 shows the particle sizes determined from the XRD results using the full width at half-maximum (FWHM) parameter. The particle size of PtRu/MWCNTs was found to be 11.97 nm.

**Figure 5:**
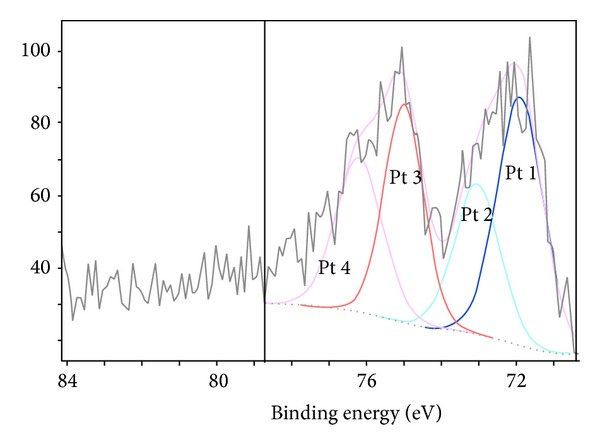
XPS analysis for Pt 4f.

The formation of the nanohybrid catalyst was characterized by XPS, as shown in Figure 5. The XPS spectra of the Co/Fe/Ni/CNT composite exhibited the Pt 4f binding energy double peak corresponding to Pt 1 at 73 eV and Pt 3 at 75 eV. Several binding energies were used to determine the elemental composition of materials bonded to Pt.  Table 2 lists Pt bonding components from [30].

**Figure 6:**
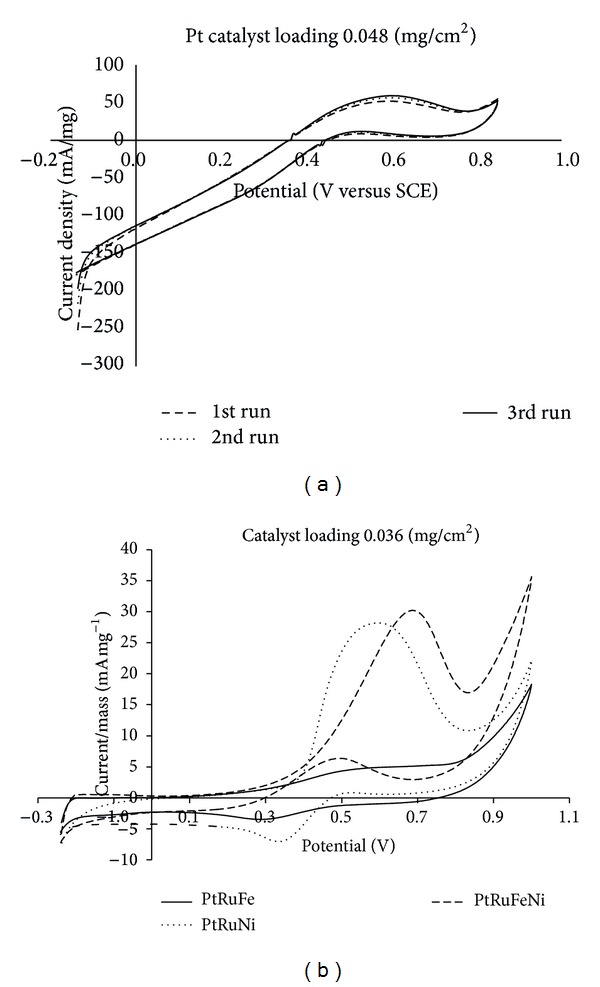
Cyclic voltammetry for MWCNT-based nanocatalyst.

Figure 6(a) shows the cyclic voltammograms for the Pt catalyst with a catalyst loading of 0.048 mg cm^−2^. A current density of 50 mA mg^−1^, which increased with each consecutive CV run, was observed for the PtRuFeNi/MWCNT nanocatalyst with a Pt loading of 0.027 mg cm^−2^_._ The PtRuFe/MWCNT, PtRuNi/MWCNT, and PtRuFeNi/MWCNT nanocatalysts were also subjected to 10 consecutive cyclic voltammetry runs. As observed in Figure 6(b), the performance of the PtRuFeNi/MWCNT catalysts, in terms of current density, was high compared to the performances of the other materials. The performance of the PtRuFe/MWCNT catalysts was the lowest of the analyzed nanocatalysts because it contained only a small amount of Pt (5%), whereas the other materials contained 15% Pt. Comparing the performance of PtRuNi/MWCNTs to PtRuFeNi/MWCNTs, it was observed that the addition of Fe^2+^ ions increased the current density. Therefore, iron and nickel additives have the potential to increase catalyst performance in DMFCs and to reduce cost [21]. Figure 6(b) shows low current density values for all samples but high potential values when a catalyst loading of 0.036 mg cm^−2^ was used. This result is attributed to the large active area of the nanocatalyst at low catalyst loading values.

Table 3 summarizes the CV results of the nanocatalysts. From this table, it is clear that the current densities of Pt/MWCNTs and PtRuFeNi/MWCNTs are comparable, even though the PtRuNiFe/MWCNTs contained a smaller amount of Pt. This result demonstrates that PtRuFeNi/MWCNTs nanocatalysts could improve the performance and kinetics of the oxidation reaction and reduce the cost of catalysts used in the membrane electrode assembly (MEA) by 15%. Similar studies used current density or mass activity as the parameter to assess the performance of DMFCs using different types of catalysts. Current density was used to determine the methanol oxidation activity based on varying catalyst compositions in half-cell configurations.  Table 4 compares the results obtained from this study with those from previous research. Finally, this study demonstrated that PtRuFeNi/MWCNTs achieved the highest current density, 31 mA mg^−1^, compared to other types of reported catalysts, namely, PtRu/MWCNTs, PtRuFe/MWCNTs, and PtRuNi/MWCNTs.

**Figure 7:**
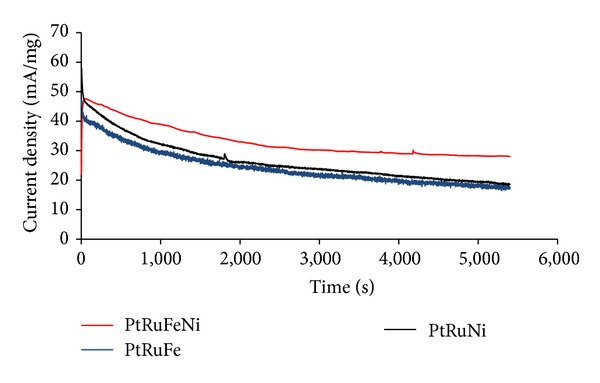
Chronoamperometry curves for (a) PtRuFeNi/MWCNT, PtRuFe/MWCNT, and PtRuNi/MWCNT in a 0.5 M H_2_SO_4_ + 1 M CH_3_OH solution.

Chronoamperometry tests were conducted to compare the long-term performance of the catalysts towards the MOR in a 0.5 M H_2_SO_4_ solution containing methanol for 5400 s.  Figure 7 shows the chronoamperometry curves for the PtRuFeNi/MWCNT, PtRuFe/MWCNT, and PtRuNi/MWCNT catalysts in 0.5 M H_2_SO_4_ + 1 M CH_3_OH at a constant potential of 0.4 V. During the initial stage, the potentiostatic current decreased rapidly for both the PtRuFe and PtRuNi catalysts, perhaps due to the formation of intermediate species, such as COads, CH_3_OHads, and CHOads, during the methanol oxidation reaction. After a long duration of operation, although the current gradually decayed for all catalysts, PtRuFeNi/MWCNT maintained a slightly higher current than the other catalysts. The long time decay can be attributed to the adsorbed SO_4_^2−^ anion on the surface of the PtRu catalysts, which can restrict the methanol oxidation reaction [29].

**Figure 8:**
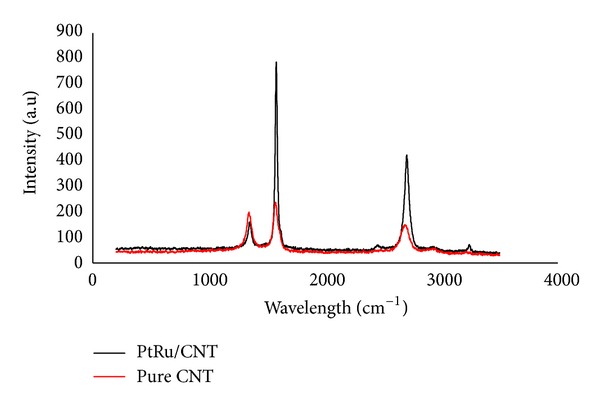
Raman spectra for PtRu/CNT and pure CNT.

In summary, CNTs coated with metal nanoparticles are gaining considerable interest for use as sensors, catalysts, and metal nanowires and for applications in nanoelectronics, such as in field-effect transistor (FET) devices. However, the attachment of metal nanoparticles to CNTs during synthesis causes distortions in the CNT geometry. In this study, MWCNTs were synthesized with Pt, Ru, Fe, and Ni using the reducing agent NaBH_4_. During reduction, –OH bonds were broken and replaced with metallic molecules. Consistent with Hyun et al. [31], the NaBH_4_ concentration was found to affect the deposition and surface composition of the prepared PtRu particles [25]. For optimal methanol electrooxidation, the molar ratio of NaBH_4_ to metal ions was determined to be 5 : 15. During synthesis, the MWCNT structures became distorted. However, this result is typical and occurs because of changes in the atomic distance of C–C bonds. This is a natural structure for CNTs. This result was confirmed by Raman spectrometry, as shown in Figure 8. The Raman emission intensity increases with the fourth power of the frequency of the source, and Raman intensities are usually directly proportional to the concentration of the active species, as shown in Figure 8. The intensity or power of a normal Raman peak has a complex dependence on the polarizability of the molecule, the intensity of the source, and the concentration of the active group, such as an attached catalyst. The change in the polarizability of a molecule during vibration is related to how easily a molecule can be deformed. This is observed in Figure 8, where the G-band spectra are located at a higher wavelength (1580 cm^−1^) than D-band (1300 cm^−1^) in the Raman spectrum which indicates the formation of PtRu/CNT. This result is due to the surface chemistry that occurs during the oxidation and synthesis process, which modifies the chemical composition of a surface by incorporating –OH onto a nanocatalyst metal.

Table 5 shows the reduction in cost for the anode catalyst presented in this study. The cost analysis was based on the amount of catalyst used for PtRu by Guo et al. [24]. It is observed that the cost of PtRuFeNi was reduced by approximately 20% based on the catalyst loading in comparison to PtRu and that it obtained a higher mass current density. The cost of the Fe, Ni catalyst and MWCNTs as a supported material did not significantly affect the total cost because they are very inexpensive compared to PTRu, as listed in Table 6. Finally, this study concluded that the PtRuFeNi catalyst introduced in this study can save up to 20% and achieves a higher mass current density than PtRu.

## 4. Conclusions

In this work, we performed two main studies: one study focused on improving the pretreatment of the catalyst support and the other focused on optimizing the synthesis of the catalyst. Based on the FT-IR results, 90°C was determined to be the optimal temperature for the pretreatment of the nanocatalyst supports. Four nanocatalysts with varying metal compositions were synthesized using a reduction process, namely, Pt/MWCNT (as a reference), PtRuFe/MWCNT, PtRuNi/MWCNT, and PtRuFeNi/MWCNT. The samples were subjected to physical and chemical analyses. Particle morphology and crystallinity were used to determine the particle size. All of the samples were nanoscale, approximately 2–30 nm, and uniform in distribution. Catalyst performance was assessed using CV in units of current density; the performance of PtRuFeNi/MWCNTs was slightly higher compared with the performance of the other nanocatalysts, and the current density was similar to that of Pt/MWCNTs. The amount of platinum in the catalyst also affected the CV results. PtRuFe/MWCNTs had the lowest current density values because this material contained less Pt than PtRuFeNi/MWCNTs. The chronoamperometry test demonstrated that, although the current gradually decayed for all the catalysts, PtRuFeNi maintained a higher current and was more stable compared to PtRuNi and PtRuFe. This result is evidence that the stability and kinetic parameters of the DMFC nanocatalyst were improved by the addition of both nickel and iron. Finally, the cost reduction analysis revealed that PtRuFeNi is capable of reducing the cost of anode catalysts for DMFCs by up to 20% compared with PtRu.

## Figures and Tables

**Table 1:** Particle sizes calculated from the XRD results.

Catalyst sample	FCC	2 Theta (°)	Full-width at half-maximum, FWHM (°)	Particle size-XRD (nm)
PtRu/MWCNT	(111)	39.814	1.089	11.97
PtRuFeNi/MWCNT	(111)	40.193	1.982	4.646
PtRuFe/MWCNT	(110)	21.597	0.274	32.02

**Table 2:** Specification of platinum 4f peaks [23].

Peak	Binding energy (eV)	Atomic mass (%)	Component
Pt 1	71.9	33.39	Pt
Pt 2	73.0	21.26	PtNi
Pt 3	75.0	25.24	Pt
Pt 4	76.2	20.1	Pt(C_32_H_16_N_8_)

**Table 3:** Summary of the cyclic voltammetry results.

Compound	Catalyst loading (mg cm^−2^)	Percent of Pt from 20% of catalyst (%)	Pt loading (mg cm^−2^)	Current density (mA mg^−1^)	Potential (V)
Pt/MWCNT	0.048	100	0.048	50	0.60
PtRuFe/MWCNT	0.036	10	0.0036	7	0.50
PtRuNi/MWCNT	0.036	75	0.027	28	0.58
PtRuNiFe/MWCNT	0.036	75	0.027	31	0.70

**Table 4:** Comparison of the current density with other studies.

Study	Item	Current density (mA mg^−1^)
This study	PtRuFeNi/MWCNT	31
Guo et al. [24]	PtRu/C	20
Jeon et al. [21]	Pt_45_Ru_45_Fe_10_/C	2.6
Jeon et al. [22]	PtRuFe/MWCNT	5.67
Liu et al. [25]	PtRuNi/C	9.0

**Table 5:** Cost reduction.

Compound	Ref.	PtRu loading (mg cm^−2^)	Fe/Ni loading (mg cm^−2^)	Current density (mA mg^−1^)	Cost reduction (%)
PtRu	[24]	0.048	—	20	—
PtRuNiFe	This study	0.027	0.0033 Ni 0.0033 Fe	31	21

**Table 6:** List of current price.

Compound	Cost per mg, USD
PtRu	0.054
Fe	4.5 × 10^−6^
Ni	7.7 × 10^−5^
MWCNT	0.0012
